# Childhood chronic stress associated with abnormal brain white matter networks in first-episode, drug-naïve MDD adolescents: a machine learning study

**DOI:** 10.3389/fpsyt.2026.1732661

**Published:** 2026-02-04

**Authors:** Zhujun Wang, Zhaoxian Ming, Meijiang Jin, Maojia Ran, Hang Zhang, Yuanmei Tao, Hanmei Xu, Shoukang Zou, Ye Wu, Li Yin

**Affiliations:** 1 Department of Psychiatry, West China Hospital of Sichuan University, Chengdu, Sichuan, China; 2 School of Computer Science and Technology, Nanjing University of Science and Technology, Nanjing, Nanjing, Jiangsu, China; 3 Frontier Science Center for Disease-related Molecular Networks, Chengdu, Sichuan, China; 4 Sichuan Clinical Medical Research Center for Mental Disorders, Chengdu, Sichuan, China

**Keywords:** adolescent, adverse childhood experience, magnetic resonance imaging, major depressive disorder, white matter

## Abstract

**Objectives:**

This study aims to identify the topological abnormalities of the brain white matter structural network in adolescents with first episode, drug-naïve major depressive disorder (MDD), and to explore the relationship between these abnormalities and chronic childhood stress.

**Methods:**

T1-weighted images and diffusion tensor imaging (DTI) data were collected from 77 first-episode, drug-naïve MDD adolescents and 31 healthy controls (HCs). A whole-brain white matter structural network was constructed for each participant. Graph-theoretical analyses were employed to investigate the topological properties of white matter structural networks. Subsequently, we applied four machine learning methods—univariate linear regression, stepwise regression, LASSO regression, and random forest—to screen for relevant variables. The selected variables were then integrated into a final multivariate linear regression model to examine their correlations with Childhood Chronic Stress Questionnaire (CCSQ) scores.

**Results:**

Compared with HCs, adolescents with MDD showed significantly larger network radius (t = -2.647, p = 0.009) and network diameter (t = -2.619, p = 0.010), as well as significantly lower small-worldness (t = 2.066, p = 0.041) and global efficiency (t = 2.083, p = 0.040) in brain white matter structural network. Local topological abnormalities were observed in multiple brain regions of adolescents with MDD, primarily involving paracentral lobular and mid cingulate cortex, MT+ complex, early auditory cortex, dorsolateral prefrontal cortex, orbital and polar frontal cortex, as well as anterior cingulate and medial prefrontal cortex. The final regression model showed that the weighted betweenness centrality in left area 1, left area 6mp, left frontal opercular area 4, left posterior OFC complex and left lateral belt complex, as well as the weighted local efficiency of the right ventral intraparietal complex and right orbital frontal complex (adjusted R^2^ = 0.270, Cohen’s f^2^ = 0.630).

**Conclusion:**

We found that chronic childhood stress is associated with local topological abnormalities in multiple regions of the white matter structural networks of adolescents with MDD. These altered white matter network topological properties may be utilized in the future to aid in identifying depressive subtypes with high childhood stress exposure.

## 1 Introduction

Major depressive disorder (MDD) is characterized by long-lasting low mood, loss of interest, and anhedonia (1). The prevalence of MDD has risen sharply in adolescents over the past decade (2). Depression in adolescents is associated with high-risk behaviors, including suicide attempts, abuse of alcohol, nicotine, and drugs (3, 4).

In recent years, neuroimaging studies have viewed MDD as a brain network dysfunction syndrome (5). Previous studies using graph theory methods have found that MDD patients exhibit alterations in brain structural and functional topological properties, including global efficiency, local efficiency, node degree, and other related network metrics (6–12). However, previous studies have primarily focused on adult patients (13–15). Notably, adolescence is a critical period for brain structural and functional development, during which the brain is highly susceptible to external environmental factors (16). Research indicates that chronic stress during childhood significantly increases the risk of developing MDD in adolescents (17). The underlying mechanisms may be related to changes in white matter integrity, as previous studies have shown that chronic stress during childhood is closely associated with axial diffusion tensor (AD), mean diffusion tensor (MD), and fractional anisotropy (FA) in individuals with MDD (18, 19). These findings deepen our understanding of the neurobiology of major depressive disorder in adolescents. However, gaps remain in our understanding of how the brain is affected by environmental factors at the network level. To the best of our knowledge, there is limited systematic research investigating the relationship between white matter networks and childhood stress specifically in adolescents with MDD.

Therefore, the primary objective of this study was to identify topological abnormalities in the white matter structural network of adolescents with MDD and to explore the association between these abnormalities and childhood chronic stress. To exclude the influence of psychiatric medications, this study included only adolescents with first-episode MDD who had not received any psychiatric medication prior to magnetic resonance imaging (MRI) scanning.

## 2 Participants and measures

This study has been approved by the Ethics Committee of West China Hospital, Sichuan University and registered on the Chinese Clinical Trial Registration Platform (ChiCTR2000033402). All participants and their guardians provided written informed consent before participating in the study.

From September 2020 to January 2022, we recruited adolescents aged 12–18 years with first-episode drug-naïve MDD from the inpatient and outpatient departments of West China Hospital, Sichuan University. Healthy controls aged 12 to 18 years were recruited through advertisements.

Adolescent MDD was diagnosed by two experienced clinical psychiatrists using the Diagnostic and Statistical Manual of Mental Disorders, Fourth Edition (DSM-IV) and the Chinese version of the Kiddie Schedules for Affective Disorders and Psychosis (KSADS-PL). All participants were required to meet the following criteria: right-handedness, completion of at least elementary school education, absence of structural brain abnormalities, and the ability to comprehend the content of the scales used in this study. Additionally, the HCs must not have any current or past psychiatric diagnoses. Participants were excluded if they had a history of electroconvulsive therapy, severe physical illness, head trauma, loss of consciousness, central nervous system disease, other DSM-IV Axis I psychiatric diagnoses, exposure to pesticides, toxins, or other substances with potential neurotoxic effects, pregnancy or breastfeeding, or any condition that was not suitable for MRI scanning.

Childhood chronic stress was assessed with the Childhood Chronic Stress Questionnaire (CCSQ), which includes three dimensions: childhood peer bullying, childhood abuse and neglect, and adverse childhood experiences (20). The questionnaire was originally developed and validated in a sample of 1,786 third- to fifth-grade students, demonstrating excellent internal consistency (Cronbach’s α = 0.946), high test-retest reliability (r = 0.921), and strong support for its hypothesized three-factor structure in confirmatory factor analysis. The Social Support Rating Scale (SSRS) was used to assess adolescents’ actual objective support, subjective support, and support utilization (21). Depression symptom was assessed using the Chinese version of the Beck Depression Inventory-II (BDI-II-C) (22).

### 2.1 MRI data acquisition

T1-weighted images and diffusion tensor imaging (DTI) were acquired using a 3.0T scanner (uMR 790, United Imaging Healthcare, Shanghai, China). Each participant’s acquisition sequence parameters are the same. T1-weighted (T1w) images were acquired using a magnetization-prepared fast gradient echo sequence: repetition time (TR)=8.4 ms; echo time (TE)=3.8 ms; flip angle=8°; slice thickness=0.8 mm; total number of sagittal slices=208; matrix size=256×256; field of view (FOV)=256×256 mm^2^; voxel size=0.8×0.8×0.8 mm^3^.

DTI data were obtained using an echo-planar imaging sequence: TR = 6.4s; TE = 0.068s; FOV = 210×210mm^2^; matrix size=140×140; slice thickness=1.5 mm; voxel size=1.5×1.5×1.5mm^3^; flip angle=90°; diffusion directions=32; b-value=1010 s/mm^2^; 98 slices.

During the scanning procedure, participants are instructed to stay calm. They are asked to remain motionless, relax with their eyes closed, and avoid falling asleep while thinking of nothing in particular. Foam pads and earplugs are used to minimize head movement and attenuate scanner noise for participants. In this procedure, images with structural abnormalities, evident head movements, and artifacts are excluded by visual inspection.

### 2.2 HCP-MMP atlas

We adopted the HCP-MMP (Human Connectome Project Multi-Modal Parcellation) atlas, which consists of 180 distinct areas per hemisphere. For organizational purposes, this atlas grouped the 180 cortical areas into 22 regions based on several criteria, including geographic proximity and functional similarities. Each region comprises a set of geographically contiguous cortical areas that share common properties based on architecture, task-fMRI profiles, and/or functional connectivity (23).

### 2.3 Image processing

We visually inspected the T1W and dMRI images of all subjects to detect any signal dropouts or artifacts. Next, we preprocessed the images via the well-established pipeline. Briefly, for both T1W and dMRI data, the procedure began with axial alignment, centering, Gibbs ringing removal based on local subvoxel shifts, and intensity inhomogeneity correction via N4ITK.

For dMRI data, we also included the following steps: (1) Marchenko–Pastur principal-component analysis (MP-PCA) denoising to improve the signal-to-noise ratio (SNR) without reducing spatial resolution; we retained 95% of the principal components to achieve an optimal balance between noise suppression and preservation of neurobiologically meaningful signal; (2) FSL’s eddy_correct tool was used for eddy current correction; (3) brain mask generation using a brain extraction tool (BET); and (4) distortion correction via registration of individual T1W and dMRI data. Finally, the transformation was applied to each diffusion-weighted volume, and the gradient vectors were rotated using the rotation matrix estimated from the affine transformation.

For dMRI data, we created the brain masks using a convolutional neural network (CNN) based segmentation tool in pnlNipype (https://github.com/pnlbwh/pnlNipype). Finally, each individual’s T1-weighted images were transformed from structural space into diffusion space through a rigid registration using FSL.

In the processing of dMRI data, the connectivity matrix was established using DSI Studio software for deterministic fiber tracking, utilizing the generalized q-space sampling imaging (GQI) reconstruction method following eddy current correction. The parameters employed for fiber tracking were as follows: (1) tracking metric - quantitative anisotropy (QA), (2) random angle threshold, (3) random tracking threshold, (4) random step size, and (5) minimum/maximum length threshold set at 30/200 mm. A total of 180 regions of interest (ROIs) were defined based on the Glasser atlas. The weight of an edge was defined as the natural logarithm of the number of fiber tracts between two brain regions.

### 2.4 Network measures

The Brain Connectivity Toolbox (URL: http://www.brain-connectivity-toolbox.net/) was used to conduct graph-theoretical analyses on connectivity matrices. This method enabled us to analyze the global properties of the brain network. Metrics evaluated included global efficiency, small-worldness, characteristic path length, network diameter, network radius, and average clustering coefficient. Furthermore, we computed some nodal properties to identify critical nodes in the brain network, specifically: nodal degree, nodal strength, local clustering coefficient, local efficiency, betweenness centrality, eigenvector centrality, and PageRank centrality.

### 2.5 Machine learning

Given the high dimensionality and complexity of topological properties in the brain’s white matter structural network, machine learning can be an effective method for analyzing such data (24). Traditional methods, such as linear regression and stepwise regression, are often limited by their inability to model nonlinear relationships. In contrast, more recent machine learning techniques, such as LASSO regression and random forests, despite their lower interpretability compared to traditional methods, offer unique advantages (25, 26). To overcome the limitations of any single method, we employed a combination of four methods—univariate linear regression, stepwise regression, LASSO regression, and random forests—to identify key variables. The results from these analyses were integrated to develop the final multivariate linear regression model, providing a comprehensive assessment of the key factors.

### 2.6 Statistical analysis

SPSS version 25 (IBM, Armonk, NY, USA) was used for statistical analyses, and p < 0.05 was considered significant for differences. The differences in age and questionnaire score were compared using independent samples t-tests. The differences in gender and family type were evaluated through chi-square tests. To determine if there were significant between-group differences in the topological metrics of the brain white matter structural network, we performed independent samples t-tests for each network topological metric. Multiple comparisons were controlled using the false discovery rate (FDR; Benjamini-Hochberg method) (corrected-P < 0.05). To quantify the practical significance of intergroup differences, we reported corresponding effect sizes. Cohen’s d is used as the effect size measure for continuous variables and all Cohen’s d values presented in the tables are reported as absolute values. For categorical variables, Cramér’s V was used as the effect size.

Subsequently, topological metrics demonstrating significant intergroup differences were included as candidate features. We employed four distinct machine learning methods—univariate linear regression, stepwise regression, LASSO regression, and random forest—to perform feature selection for associations with clinical outcomes (CCSQ and SSRS total scores). Age, gender, group, education level and total intracranial volume (TIV) were included as covariates in all models. To improve model interpretability, we further assessed feature importance within each model to quantify the relative contribution of each variable to CCSQ total scores and SSRS total scores. For the random forest model, we used 10-fold cross-validation to select the optimal feature-importance threshold (0.1-0.95) that minimizes mean squared error (MSE). In each cross-validation iteration, the data were partitioned into 10 subsets: 9 for training and 1 for validation. We then integrated the results from the four methods. Variables were retained if they met any of the following criteria: (1) significant univariate association (p < 0.05), (2) selection by stepwise regression, (3) non-zero coefficient in LASSO regression, or (4) surpassing the cross-validated importance threshold in random forest. After merging outcomes, removing duplicates, and adjusting for pre-included covariates, a final set of brain network variables was derived.

Using this final set of variables, combined with all original covariates (age, gender, group, education level and TIV), we constructed a multiple linear regression model, and applied the Akaike Information Criterion (AIC) to identify the well-fitting model. We evaluated the final model using the overall goodness of fit (R², adjusted R²), regression coefficients with corresponding p-values, and the complete regression equation.

## 3 Results

### 3.1 Demographic and clinical characteristics

The demographic and clinical characteristics of all participants are shown in **Table 1**. The final analysis included 77 adolescents with MDD (mean age 14.27 [1.38] years; 18 males [23.4%]) and 31 HCs (mean age 14.00 [1.51] years; 13 males [41.9%]). No statistically significant differences were found in age, gender or educational level between HCs and MDD patients. There were significant differences between the two groups in family type (χ² = 11.163, p = 0.008). Compared with HCs, MDD patients had significantly higher CCSQ scores on the total scale (t = 3.363, p = 0.001) and all subscales (childhood peer bullying: t = 2.702, p = 0.008; childhood maltreatment and neglect: t = 2.532, p = 0.013; adverse childhood experiences: t = 4.100, p < 0.001). Conversely, the MDD group had significantly lower SSRS scores on the total scale (t = -7.972, p < 0.001) and all subscales compared with the HCs (objective support: t = -4.014, p < 0.001; subjective support: t = -5.484, p < 0.001; utilization of support: t = -4.745, p < 0.001).

**Table 1:** Demographic and clinical characteristics of adolescents with MDD and HCs.

Characteristics	MDD (n=77)	HCs (n=31)	χ2/t/Z	p-value	Effect size
Demographic characteristics					
**Male** (N, %)	18 (23.4%)	13 (41.9%)	3.720	0.054	0.163
**Age**, years (mean [SD])	14.27 [1.38]	14.00 [1.51]	0.904	0.368	
**Education**			1.738	0.391	0.126
Elementary School (N, %)	3 (3.9%)	1 (3.2%)			
Middle School (N, %)	47 (61.0%)	23 (74.2%)			
High School (N, %)	27 (35.1%)	7 (22.6%)			
**Family type**			11.163	0.008	0.338
Nuclear family (N, %)	31 (41.9%)	21 (67.7%)			
Extended family (N, %)	19 (25.7%)	9 (29.0%)			
Live without their parents (N, %)	6 (8.1%)	0 (0.0%)			
Single Parent Family (N, %)	18 (24.3%)	1 (3.2%)			
Clinical characteristics					
**BDI-II-C scores** (mean [SD])	34.41 [10.83]	9.26 [9.17]	11.383	<0.001	2.421
**CCSQ scores** (mean [SD])	115.42 [58.88]	87.63 [26.85]	3.363	0.001	0.536
Childhood peer bullying (mean [SD])	25.3 [15.62]	19.45 [6.85]	2.702	0.008	0.426
Childhood maltreatment and neglect (mean [SD])	50.23 [27.17]	40.23 [13.67]	2.532	0.013	0.414
Adverse childhood experiences (mean [SD])	38.47 [18.55]	27.18 [9.85]	4.100	<0.001	0.682
**SSRS scores** (mean [SD])	27.21 [4.38]	35.14 [5.35]	-7.972	<0.001	1.696
objective support (mean [SD])	6.44 [1.58]	7.72 [1.25]	-4.014	<0.001	0.857
subjective support (mean [SD])	14.91 [3.09]	18.58 [3.31]	-5.484	<0.001	1.164
support utilization (mean [SD])	5.70 [1.52]	7.84 [2.32]	-4.745	<0.001	1.200

MDD, major depressive disorder; HCs, healthy controls; BDI, Beck Depression Inventory; CCSQ, Childhood Chronic Stress Questionnaire; SSRS, Social Support Rating Scale; Statistical notations: N, number of participants; %, percentage.

Effect sizes for continuous variables are reported as Cohen’s d; those for categorical variables are reported as Cramér’s V.

### 3.2 Network metrics

#### 3.2.1 Between-group differences in global topological metrics

**Figure 1:**
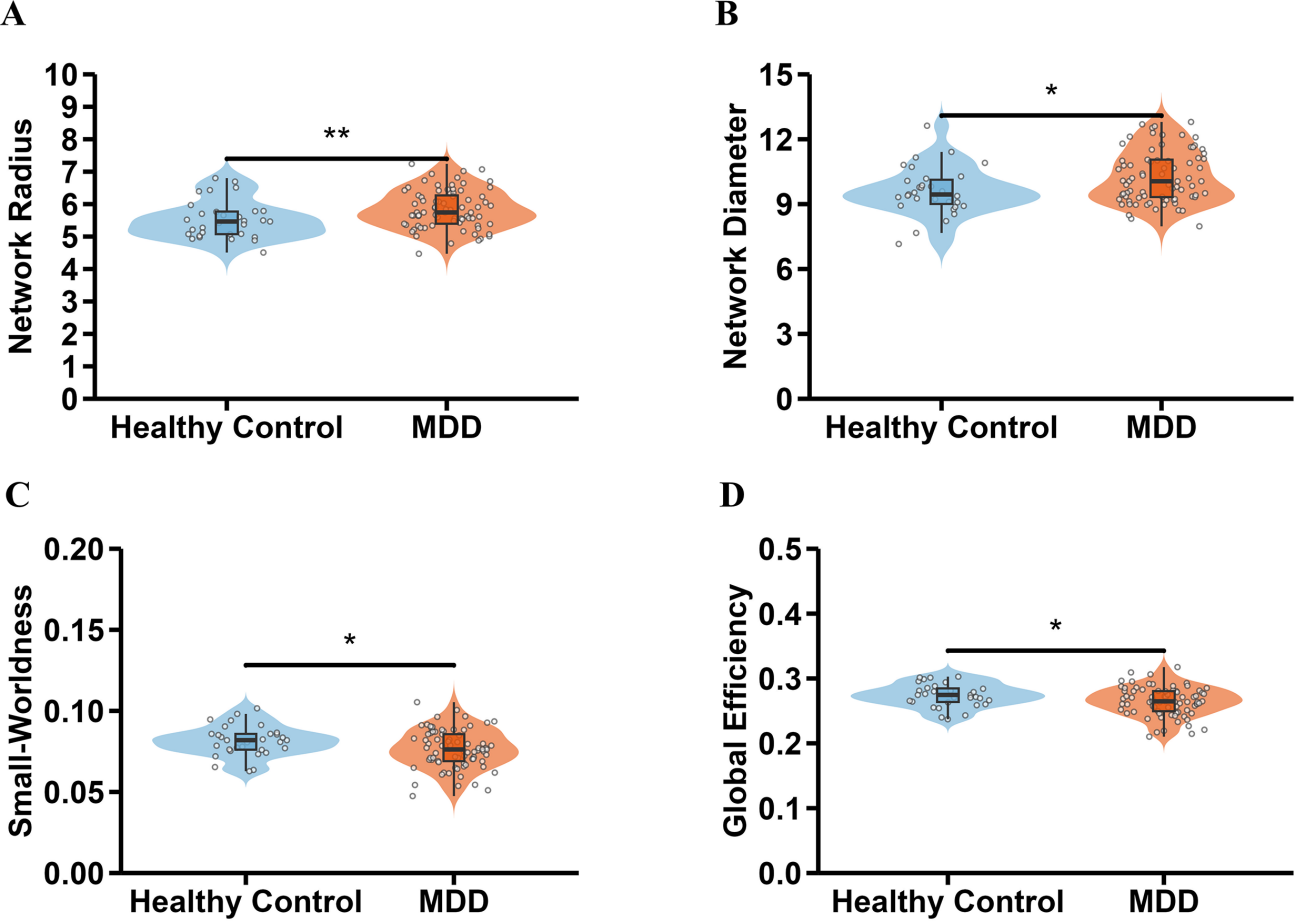
Significant between-group differences in global metrics **(A)** Significant between-group differences in network radius. **(B)** Significant between-group differences in network diameter. **(C)** Significant between-group differences in small-worldness. **(D)** Significant between-group differences in global efficiency. ∗p < 0.05, ∗∗p < 0.01.

Compared with HCs, the MDD group showed significantly higher network radius (t = 2.647, p = 0.009) and network diameter (t = 2.619, p = 0.010) and had significantly lower small-worldness (t = -2.066, p = 0.041) and global efficiency (t = -2.083, p = 0.040) (**Figure 1**). No significant differences were observed between the two groups in characteristic path length or average clustering coefficient (all p>0.05).

#### 3.2.2 Between-group differences in local topological metrics

**Figure 2:**
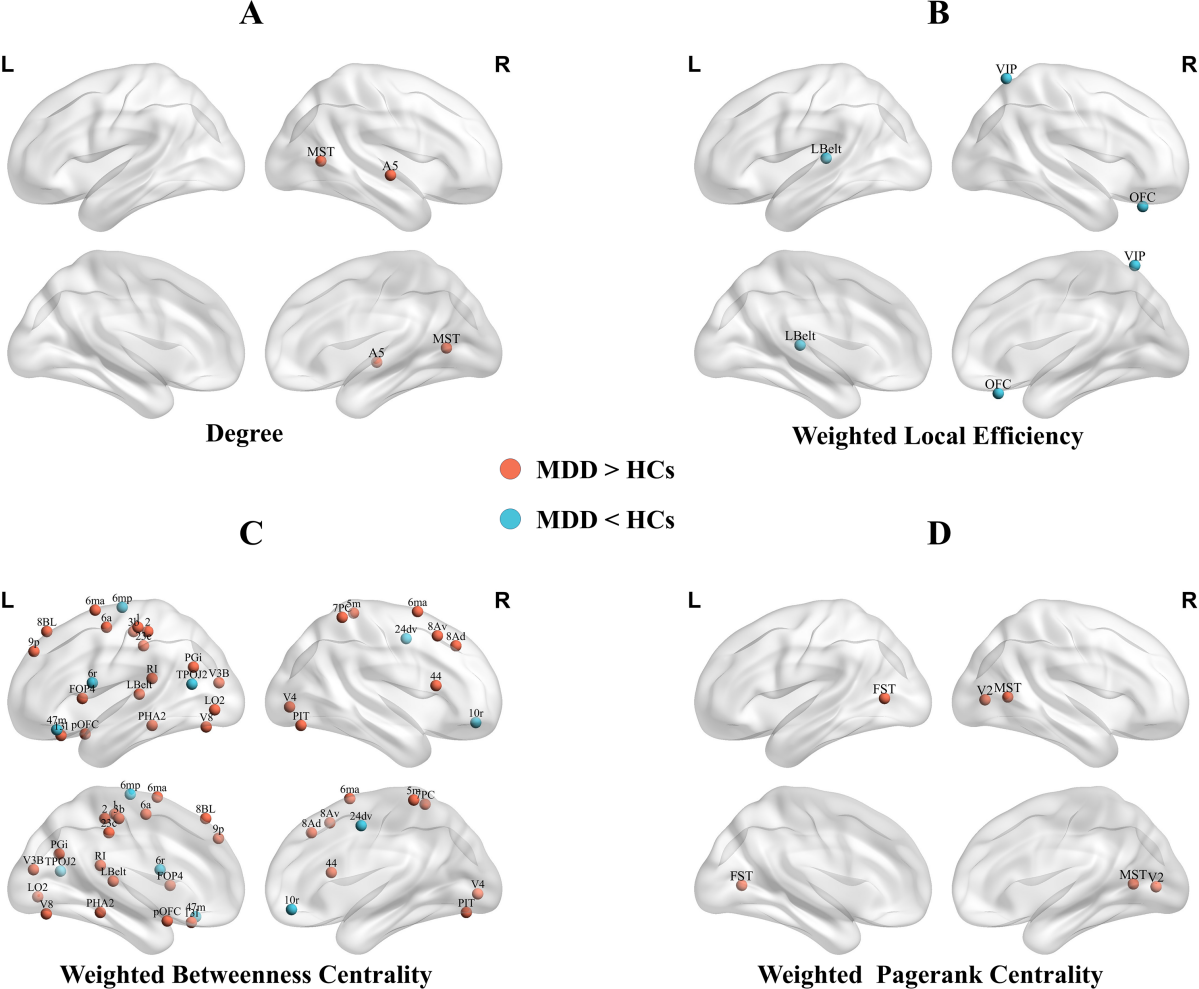
Brain regions showed significantly higher nodal degree **(A)**, lower weighted betweenness centrality **(B)**, altered weighted betweenness centrality **(C)**, and higher weighted PageRank centrality **(D)** in adolescents with MDD compared with HCs. MDD, major depressive disorder; HCs, health controls;R, right;L, left;MST, Medial Superior Temporal Area; A5, Auditory 5 Complex; Lbelt, Lateral Belt Complex; VIP, Ventral IntraParietal Complex; OFC, Orbital Frontal Complex; 6mp, Area 6mp; 47m, Area 47m; 6r, Rostral Area 6; TPOJ2, Area TemporoParietoOccipital Junction 2; 24dv, Ventral Area 24d; 10r, Area 10r; V8, Eighth Visual Area; V3B, Area V3B; 6ma, Area 6m anterior; 1, Area 1; 2, Area 2; 6a, Area 6 anterior; PGi, Area PGi; PHA2, ParaHippocampal Area 2; pOFC, posterior OFC Complex; V4, Fourth Visual Area; 3b, Primary Sensory Cortex; LO2, Area Lateral Occipital 2; 23c, Area 23c; 8BL, Area 8B Lateral; 9p, Area 9 Posterior; 13l, Area 13l; RI, RetroInsular Cortex; FOP4, Frontal Opercular Area 4; PIT, Posterior InferoTemporal Complex; 5m, Area 5m; 7PC, Area 7PC; 8Av, Area 8Av; 8Ad, Area 8Ad; 44, Area 44; FST, Area FST; V2, Second Visual Area.

Significant differences in local topological metrics between adolescents with MDD and HCs are shown in **Figure 2**; **Table 2** (all p < 0.05, FDR-corrected).

**Table 2:** Differences in local topological metrics between MDD patients and HCs.

Metric	Regions	Cortical sections	t	p Value	Cohen’s d
Degree					
MDD > HCs					
	Right Medial Superior Temporal Area	5	4.115	0.027	0.669
	Right Auditory 5 Complex	11	3.936	0.027	0.633
Weighted local efficiency					
MDD < HCs					
	Left Lateral Belt Complex	10	-4.735	0.001	0.753
	Right Ventral IntraParietal Complex	16	-3.689	0.043	0.785
	Right Orbital Frontal Complex	20	-5.283	<0.001	0.789
Weighted betweenness centrality					
MDD < HCs					
	Left Area 6mp	7	-3.209	0.033	0.978
	Left Area 47m	20	-3.284	0.032	1.045
	Left Rostral Area 6	8	-3.201	0.033	0.891
	Left Area TemporoParietoOccipital Junction 2	15	-3.181	0.033	0.804
	Right Ventral Area 24d	7	-3.352	0.028	1.041
	Right Area 10r	19	-2.949	0.046	0.799
MDD > HCs					
	Left Eighth Visual Area	4	3.893	0.004	0.622
	Left Area V3B	3	3.869	0.004	0.559
	Left Area 6m anterior	7	4.014	0.003	0.614
	Left Area 1	6	3.422	0.015	0.587
	Left Area 2	6	4.003	0.003	0.614
	Left Area 6 anterior	8	2.852	0.046	0.465
	Left Area PGi	17	3.481	0.012	0.561
	Left ParaHippocampal Area 2	13	4.581	0.001	0.622
	Left posterior OFC Complex	19	2.910	0.042	0.482
	Left Lateral Belt Complex	10	3.208	0.026	0.537
	Left Primary Sensory Cortex	6	2.819	0.046	0.462
	Left Area Lateral Occipital 2	5	4.510	0.001	0.676
	Left Area 23c	7	2.821	0.046	0.600
	Left Area 8B Lateral	22	3.641	0.008	0.533
	Left Area 9 Posterior	22	4.374	0.001	0.623
	Left Area 13l	20	3.033	0.033	0.445
	Left RetroInsular Cortex	10	3.017	0.033	0.454
	Left Frontal Opercular Area 4	12	4.746	0.001	0.754
	Right Fourth Visual Area	2	4.299	0.001	0.647
	Right Area 6m anterior	7	3.640	0.008	0.549
	Right Posterior InferoTemporal Complex	4	2.965	0.037	0.482
	Right Area 5m	7	5.259	<0.001	0.746
	Right Area 7PC	16	2.863	0.046	0.477
	Right Area 8Av	22	3.313	0.019	0.525
	Right Area 8Ad	22	4.574	0.001	0.663
	Right Area 44	21	4.017	0.003	0.565
Weighted pagerank centrality					
MDD > HCs					
	Left Area FST	5	4.616	0.004	0.739
	Right Medial Superior Temporal Area	5	3.743	0.040	0.665
	Right Second Visual Area	2	3.795	0.040	0.607
Weighted eigenvector centrality					
MDD > HCs					
	Right posterior OFC Complex	19	4.914	0.001	0.726

The p-value was adjusted for multiple comparisons using the Benjamini-Hochberg FDR correction method.

The region is assigned to one of the 22 cortical sections defined by the HCP-MMP parcellation (23): 1, Primary Visual Cortex(V1); 2, Early Visual Cortex; 3, Dorsal Stream Visual Cortex; 4, Ventral Stream Visual Cortex; 5, MT+ Complex and Neighboring Visual Areas; 6, Somatosensory and Motor Cortex; 7, Paracentral Lobular and Mid Cingulate Cortex; 8, Premotor Cortex; 9, Posterior Opercular Cortex; 10, Early Auditory Cortex; 11, Auditory Association Cortex; 12, Insular and Frontal Opercular Cortex; 13, Medial Temporal Cortex; 14, Lateral Temporal Cortex; 15, Temporo-Parieto-Occipital Junction; 16, Superior Parietal Cortex; 17, Inferior Parietal Cortex; 18, Posterior Cingulate Cortex; 19, Anterior Cingulate and Medial Prefrontal Cortex; 20, Orbital and Polar Frontal Cortex; 21, Inferior Frontal Cortex; 22, DorsoLateral Prefrontal Cortex.

##### 3.2.2.1 Nodal degree

Adolescents with MDD showed significantly higher nodal degree in the right medial superior temporal area (MT+ Complex) and right auditory 5 complex (Association Auditory Cortex) compared with HCs.

##### 3.2.2.2 Weighted local efficiency

MDD participants showed significantly lower local efficiency in the left lateral belt complex (Early Auditory Cortex), right ventral intraparietal complex (Superior Parietal Cortex), and right orbital frontal complex (Orbital Frontal Cortex).

##### 3.2.2.3 Weighted betweenness centrality

Widespread alterations in weighted betweenness centrality were observed. Compared with HCs, adolescents with MDD exhibited higher weighted betweenness centrality mainly in left area 6m anterior, left area 23c, right area 6m anterior, and right area 5m(Paracentral Lobular and Mid Cingulate Cortex); left area 8B lateral, left area 9 posterior, right area 8Av, and right area 8Ad(DorsoLateral Prefrontal Cortex); left area 1, left area 2 and left primary sensory cortex(Somatosensory and Motor Cortex); left eighth visual area and right posterior inferotemporal complex(Ventral Stream Visual Cortex); and left lateral belt complex and left retroinsular cortex(Early Auditory Cortex).

Conversely, adolescents with MDD showed significantly lower weighted betweenness centrality mainly in the left area 6mp and right ventral area 24d (Paracentral Lobular and Mid Cingulate Cortex); left rostral area 6(Premotor Cortex); left area temporoparietooccipital junction 2(Temporo-Parieto-Occipital Junction); right area 10r (Anterior Cingulate and Medial Prefrontal Cortex); and left area 47m (Orbital and Polar Frontal Cortex).

##### 3.2.2.4 Weighted pagerank centrality

Compared with HCs, adolescents with MDD exhibited significantly higher weighted PageRank centrality in the left area FST and right medial superior temporal area (MT+ complex) and right second visual area (Early Visual Cortex).

##### 3.2.2.5 Weighted eigenvector centrality

Compared with HCs, adolescents with MDD exhibited significantly higher weighted eigenvector centrality in the right posterior OFC complex (Anterior Cingulate and Medial Prefrontal Cortex) (**Supplementary Figure S1**).

##### 3.2.2.6 Other local topological metrics

No significant differences were observed in nodal strength or local clustering coefficient between the two groups (both p > 0.05, FDR-corrected).

Although sex ratio did not differ significantly (p = 0.054), we tested for sex-by-diagnosis interactions in all significant metrics. None survived correction, supporting the generalizability of findings across sexes (eMethods, **Supplementary Table S7**).

### 3.3 Machine learning

Utilizing four distinct modeling techniques, we selected a set of variables potentially linked to the clinical outcomes (**Supplementary Tables S2**–**S5**).

**Figure 3:**
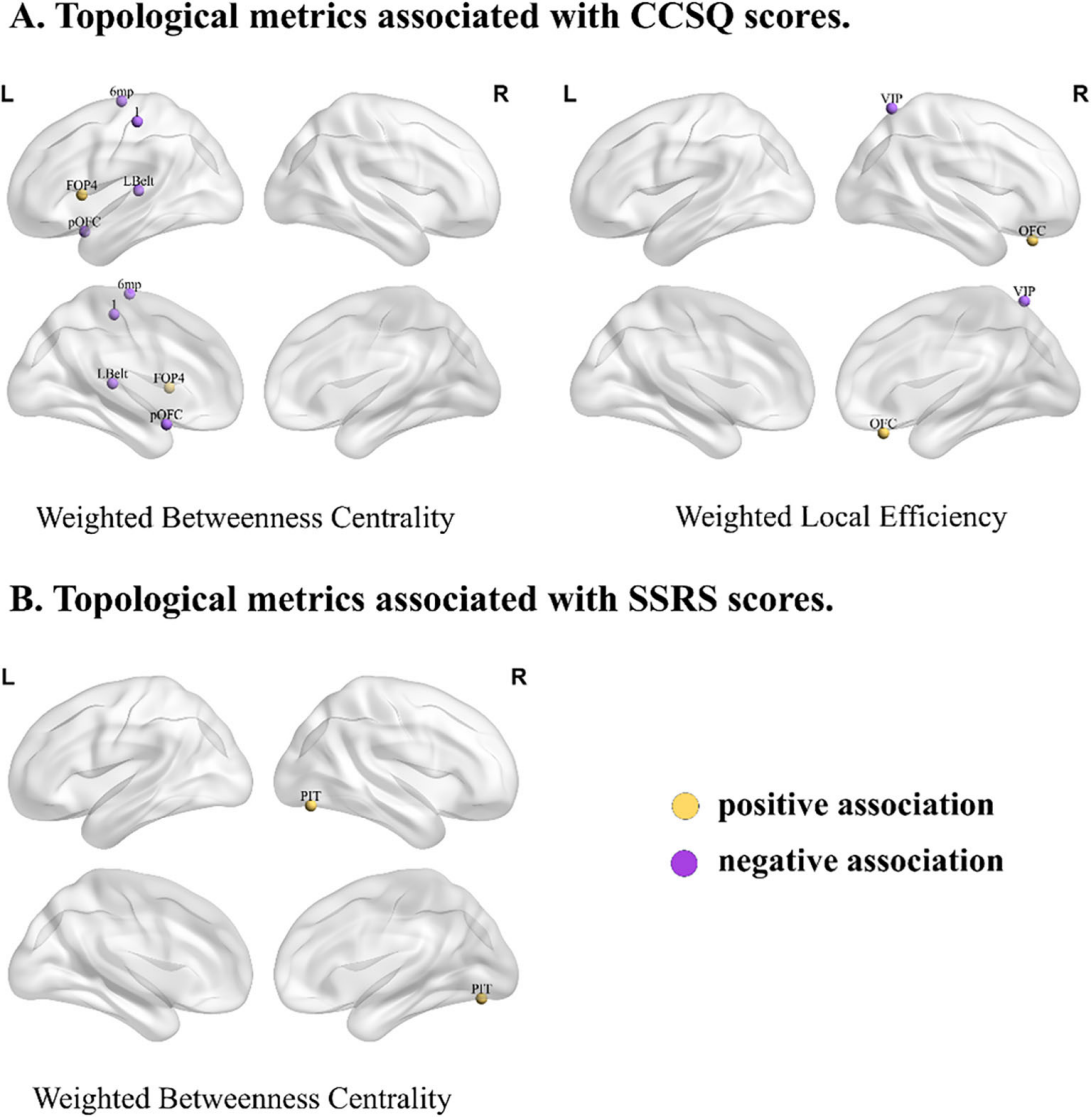
Topological metrics associated with CCSQ and SSRS scores. **(A)** Topological metrics associated with CCSQ. **(B)** Topological metrics associated with SSRS scores. Nodes colored yellow represent brain regions where topological metrics are positively associated with CCSQ or SSRS scores. Nodes colored purple represent brain regions where topological metrics are negatively associated with CCSQ or SSRS scores. Abbreviations of cortical regions: VIP, Ventral IntraParietal Complex (Superior Parietal Cortex); OFC, Orbital Frontal Complex (Orbital and Polar Frontal Cortex); 1, area 1(Somatosensory and Motor Cortex); 6mp, Area 6mp (Paracentral Lobular and Mid Cingulate Cortex); FOP4, Frontal Opercular Area 4 (Insular and Frontal Opercular Cortex); pOFC, posterior OFC Complex (Anterior Cingulate and Medial Prefrontal Cortex); Lbelt, Lateral Belt Complex(Early Auditory Cortex); PIT, Posterior InferoTemporal Complex (Ventral Stream Visual Cortex).

In the final regression model(**Supplementary Table S6**), CCSQ total scores were significantly associated with the following network metrics: weighted betweenness centrality in left area 1 (1; β = -16.830, p<0.001), left area 6mp (6mp; β = -11.281, p = 0.034), left frontal opercular area 4 (FOP4; β = 10.973, p = 0.031), left posterior OFC complex (pOFC; β = -11.222, p = 0.021) and left lateral belt complex (Lbelt; β = -13.828, p = 0.004); and the weighted local efficiency of the right ventral intraparietal complex (VIP; β = -13.233, p = 0.010)and right orbital frontal complex (OFC; β = 13.345, p = 0.009) (**Figure 3**). The final model demonstrated a large effect size (Cohen’s f^2^ = 0.630) and accounted for 27.0% of the variance in the outcome after adjusting for the number of predictors (adjusted R^2^ = 0.270). Higher betweenness centrality in left area 1, left 6mp, left pOFC and left LBelt, as well as higher local efficiency in right VIP, was associated with lower CCSQ scores. In contrast, higher weighted betweenness centrality in left FOP4 and local efficiency in right OFC were associated with higher CCSQ scores.

The final model for SSRS total scores demonstrated a huge effect size (Cohen’s f² = 1.002) and accounted for 41.4% of the variance in the outcome (adjusted R² = 0.414). Significant predictors included group (β = –7.184, p < 0.001), gender (β = 2.698, p = 0.019), and weighted betweenness centrality of the right posterior inferotemporal complex (PIT; β = 1.582, p = 0.003) (**Figure 3**). Higher weighted betweenness centrality in the right PIT was associated with higher SSRS total scores.

## 4 Discussion

Adolescence is a critical transitional period shaped by the interplay of biological, psychological, and social factors (27), during which the prevalence of MDD increases sharply (2). This study investigated alterations in white matter network topology among adolescents with MDD and their association with childhood chronic stress. Our study focused on first-episode, drug-naive adolescents with MDD. These individuals were experiencing their initial depressive episode and had not yet commenced any form of treatment before MRI scanning, thereby excluding the influence of medication on brain function.

Our study has identified local topological properties associated with chronic childhood stress in adolescents with MDD, located within the early auditory cortex and secondary visual networks. Choi et al. found that individuals who witnessed domestic violence during childhood exhibited reduced white matter integrity in the visual limbic pathway by early adulthood (28). Exposure to parental verbal abuse was associated with increased gray matter volume in the left superior temporal gyrus (auditory cortex) (29). Prolonged childhood exposure to adversities such as domestic conflict and verbal abuse may reshape the brain’s primary processing of auditory and visual information. While these adaptive changes may aid in recognizing environmental dangers in the short term, they lead individuals to unconsciously prioritize and amplify negative stimuli over time (30). This selective focus on negative stimuli lays the groundwork for the development of depression (31).

Beyond sensory areas, we found that chronic childhood stress was significantly associated with local topological properties in multiple key nodes of the prefrontal cortex (left pOFC, left FOP4, and right OFC). Chronic stress can activate the hypothalamic-pituitary-adrenal (HPA) axis, elevating glucocorticoid levels. Glucocorticoids act on brain regions that express high levels of glucocorticoid receptors, such as the prefrontal cortex, and trigger oxidative stress (32). This oxidative stress impairs oligodendrocyte precursor cell differentiation and disrupts myelination (33), ultimately diminishing white matter microstructural integrity (34). The topological abnormalities observed in our study may therefore represent the macroscopic network-level consequences of this underlying neurobiological pathway.

In this study, we found that adolescents with MDD exhibited higher network radius and diameter, along with lower small-worldness and global efficiency. These altered metrics converge to indicate that the white matter structural networks in MDD adolescents demonstrate elongated communication pathways, diminished global efficiency and integrative capacity. Our findings are consistent with previous studies in adults with MDD (35, 36), but contrast with those observed in first-episode, drug-naïve adults. Previous work in this latter group has reported increased global efficiency and reduced characteristic path length (37), suggesting enhanced information transmission within the brain’s structural network. These findings may indicate that adolescence represents a critical developmental window during which white matter networks are particularly vulnerable. In contrast, the mature adult brain retains greater compensatory capacity and short-term adaptive plasticity despite initial depressive episodes.

However, we observed no significant differences in characteristic path length and clustering coefficient between the MDD and HCs. This might be because the first-episode, drug-naïve MDD patients recruited in this study were in the early stages of the disease, with some global topological properties yet to be disrupted (38). These two global topological metrics have also shown heterogeneity in previous studies. Wu et al. found no significant changes in characteristic path length but a significant decrease in clustering coefficient in the white matter structural networks of MDD patients compared to healthy controls (13); Li et al. observed no significant change in clustering coefficient in the white matter structural networks of MDD patients (39); Xu et al. found increased characteristic path length and decreased clustering coefficient in the white matter structural networks of MDD patients compared to healthy controls (40). The inconsistent findings across studies regarding path length and clustering coefficient likely stem from the high heterogeneity of depression. For instance, He et al. further subdivided depressed patients into major depressive disorder with melancholic features (M-MDD) and non-melancholic MDD (NM-MDD), revealing significant differences in WM network alterations between the two MDD groups (41); Another study revealed significant differences in white matter structural network properties among MDD patients with different onset ages (6). Therefore, future research should focus on refining depression subtypes to enhance the sensitivity and specificity of neuroimaging biomarkers.

Additionally, we investigated differences in local topological metrics of brain white matter structural networks between adolescents with MDD and healthy controls. In graph-theoretical analyses, node degree, local efficiency, and centrality are key metrics for evaluating the significance and impact of individual nodes within a network. In this study, we observed alterations in local topological properties across several brain regions in MDD adolescents, including the frontal, parietal, and occipital lobe, as well as lateral occipitotemporal regions. These regions predominantly participate in secondary visual, somatomotor, default mode, cingulo-opercular, and frontoparietal networks (according to Cole-Anticevic’s brain network parcellation (42)). These networks are critically involved in key neurocognitive processes, including visual information processing (43, 44), motor coordination (11, 45), introspection and self-referential thought (46), and cognitive control (47–49). Dysfunction within these network nodes may underlie core clinical features of MDD, such as cognitive biases, psychomotor retardation, rumination, and impaired emotional regulation (2). Therefore, our research support the idea of adolescent MDD as a syndrome of network dysfunction.

However, this study also has some limitations. First, given the relatively young age of the participants, we cannot exclude the possibility that some patients may be diagnosed with bipolar disorder or schizophrenia in the future. Second, the relatively small sample size and imbalance between groups may have reduced statistical power, thereby limiting the robustness and generalizability of the findings. Furthermore, as a cross-sectional study, this research cannot establish causal relationships between brain network topological properties and chronic stress or MDD. Although we identified abnormal white matter structural network topologies in adolescent MDD, it remains unclear whether this abnormality represents a neurophysiological risk factor preceding the onset of depression or a vulnerability marker for childhood chronic stress. Future research could address this gap by assessing developmental changes in brain structural networks across longitudinal samples. Lastly, the use of self-reported questionnaires in this study may be subject to recall bias, which could affect the accuracy of the reported information. To further elucidate the pr) and role of neural circuits in the pathogenesis of adolescent depression, future research should incorporate larger and more matched samples, employ multimodal neuroimaging techniques (e.g., fMRI, PET), and examine both structural and functional brain networks. Moreover, dynamic brain network analyses may provide novel insights into how time-varying topological properties relate to fluctuations in depressive symptoms over time.

## 5 Conclusion

In this study, we identified an impaired topology of the brain white matter structural network in adolescents with MDD. We found that chronic childhood stress is associated with local topological abnormalities across multiple regions of the white matter structural networks of adolescents with MDD, including the secondary visual, auditory, and orbito-affective networks. This pattern suggests vulnerability in these regions among adolescents with MDD who experienced chronic childhood stress. These altered white matter network topological properties may be utilized in the future to aid in identifying depressive subtypes with high childhood stress exposure, thereby guiding targeted social support interventions or individualized psychotherapy strategies. Moreover, they could also be leveraged to guide the selection of targets for non-invasive neuromodulation techniques—such as transcranial magnetic stimulation (TMS)—enabling more precise, individualized neural circuit-based interventions.

## Data Availability

The raw data supporting the conclusions of this article will be made available by the authors, without undue reservation.
