# Supplementary material for: Childhood chronic stress associated with abnormal brain white matter networks in first-episode, drug-naïve MDD adolescents: a machine learning study
Source: Front Psychiatry. 2026 Feb 4;17:1732661. doi: 10.3389/fpsyt.2026.1732661 (PMC12913395; doi:10.3389/fpsyt.2026.1732661)
Supplement: Supplementary file 1 [file Supplementaryfile1.docx]

Table of Contents

[eMethods 2](#_Toc220449522)

[Table S1. Differences in global topological metrics between MDD patients and HCs 2](#_Toc220449523)

[Table S2. The 5 Most Important Predictor Variables in Univariate Linear Regression Analysis 2](#_Toc220449524)

[Table S3. The results of the stepwise regression analysis 2](#_Toc220449525)

[Table S4. The results of LASSO regression analysis 3](#_Toc220449526)

[Table S5. Top 5 variables by importance in the random forest model 3](#_Toc220449527)

[Table S6. Final multiple linear regression model for CCSQ and SSRS scores 3](#_Toc220449528)

[Table S7. Group × sex interaction effects on brain network metrics 4](#_Toc220449529)

[Figure S1. The brain region showed significantly higher weighted eigenvector centrality in adolescents with MDD compared with HCs. 5](#_Toc220449530)

### eMethods

Given the group difference in gender distribution (p = 0.054), we conducted a post-sensitivity analysis to examine whether the observed brain network alterations were moderated by gender. Specifically, we reran all models for the 45 initially significant network metrics, including an additional “group × gender” interaction term, while controlling for age, education, and family type. Multiple comparisons were corrected using the Benjamini–Hochberg procedure (FDR, q = 0.05).”

### Table S1. Differences in global topological metrics between MDD patients and HCs

| **Metric** | **t** | **p value** | **Cohen's d** |
| --- | --- | --- | --- |
| Small-Worldness | -2.066 | 0.041 | 0.440 |
| Global Efficiency | -2.083 | 0.040 | 0.443 |
| Network Diameter | 2.619 | 0.010 | 0.557 |
| Network Radius | 2.647 | 0.009 | 0.563 |
| Characteristic Path Length | 1.801 | 0.075 | 0.383 |
| Average Clustering Coefficient | -1.493 | 0.139 | 0.269 |

### Table S2. The 5 Most Important Predictor Variables in Univariate Linear Regression Analysis

| **Dependent Variable** | **Variables** | **R²** | **p value** |
| --- | --- | --- | --- |
| **CCSQ** | weighted local efficiency of R_VIP | 0.060 | 0.011 |
|  | weighted betweenness centrality of L_9p | 0.059 | 0.011 |
|  | group | 0.056 | 0.013 |
|  | weighted betweenness centrality of L_RI | 0.049 | 0.021 |
|  | weighted betweenness centrality of R_44 | 0.044 | 0.030 |
| **SSRS** | group | 0.375 | <0.001 |
|  | weighted betweenness centrality of R_24dv | 0.121 | <0.001 |
|  | weighted betweenness centrality of L_6mp | 0.110 | <0.001 |
|  | weighted local efficiency of L_LBelt | 0.088 | 0.002 |
|  | network radius | 0.077 | 0.004 |

Abbreviations: CCSQ, Childhood Chronic Stress Questionnaire；SSRS, Social Support Rating Scale; R, right；L, left；VIP, Ventral IntraParietal Complex; 9p, Area 9 Posterior; RI, RetroInsular Cortex; 44, Area 44; 24dv, Ventral Area 24d; 6mp, Area 6mp; Lbelt, Lateral Belt Complex.

### Table S3. The results of the stepwise regression analysis

| **Dependent Variable** | **Adjusted R²** | **Cohen's f^2^** | **RMSE** | **Significant Variables（p<0.05）** |
| --- | --- | --- | --- | --- |
| **CCSQ** | 0.184 | 0.286 | 47.998 | group |
|  |  |  |  | weighted local efficiency of R_OFC |
|  |  |  |  | weighted betweenness centrality of L_pOFC |
|  |  |  |  | weighted betweenness centrality of L_LBelt |
| **SSRS** | 0.433 | 0.850 | 4.434 | group |
|  |  |  |  | weighted betweenness centrality of R_V4 |
|  |  |  |  | weighted betweenness centrality of R_PIT |

Abbreviations: CCSQ, Childhood Chronic Stress Questionnaire；SSRS, Social Support Rating Scale; R, right；L, left；OFC, Orbital Frontal Complex; pOFC, posterior OFC Complex; Lbelt, Lateral Belt Complex; V4, Fourth Visual Area; PIT, Posterior InferoTemporal Complex.

### Table S4. The results of LASSO regression analysis

| **Dependent Variable** | **Adjusted R²** | **Cohen's f^2^** | **Significant Variables（p<0.10）** |
| --- | --- | --- | --- |
| **CCSQ** | 0.029 | 0.102 | group |
|  |  |  | age |
| **SSRS** | 0.391 | 0.870 | group |
|  |  |  | gender |
|  |  |  | weighted betweenness centrality of L_6mp |
|  |  |  | weighted betweenness centrality of R_24dv |

Abbreviations: CCSQ, Childhood Chronic Stress Questionnaire；SSRS, Social Support Rating Scale; R, right；L, left; 6mp, Area 6mp; 24dv, Ventral Area 24d.

### Table S5. Top 5 variables by importance in the random forest model

| **Dependent Variable** | **Variables** | **Importance** |
| --- | --- | --- |
| **CCSQ** | weighted betweenness centrality of R_24dv | 10.662 |
|  | weighted local efficiency of R_VIP | 7.3298 |
|  | weighted betweenness centrality of L_FOP4 | 7.1167 |
|  | weighted betweenness centrality of L_9p | 6.9712 |
|  | weighted betweenness centrality of L_RI | 5.5498 |
| **SSRS** | group | 0.394 |
|  | weighted betweenness centrality of R_24dv | 0.085 |
|  | weighted betweenness centrality of L_6mp | 0.070 |
|  | weighted betweenness centrality of L_6a | 0.056 |
|  | weighted betweenness centrality of L_6r | 0.054 |

Abbreviations: CCSQ, Childhood Chronic Stress Questionnaire；SSRS, Social Support Rating Scale; R, right；L, left; 24dv, Ventral Area 24d; VIP, Ventral IntraParietal Complex; FOP4, Frontal Opercular Area 4; 9p, Area 9 Posterior; RI, RetroInsular Cortex; 6mp, Area 6mp; 6a, Area 6 anterior; 6r, Rostral Area 6.

### Table S6. Final multiple linear regression model for CCSQ and SSRS scores

| **Dependent Variables** | **Adjusted R²** | **Cohen's f^2^** | **Significant Variables** | **β** | **p value** |
| --- | --- | --- | --- | --- | --- |
| **CCSQ** | 0.270 | 0.630 | weighted local efficiency of R_VIP | -13.233 | 0.010 |
|  |  |  | weighted local efficiency of R_OFC | 13.345 | 0.009 |
|  |  |  | weighted betweenness centrality of L_1 | -16.830 | <0.001 |
|  |  |  | weighted betweenness centrality of L_6mp | -11.281 | 0.034 |
|  |  |  | weighted betweenness centrality of L_FOP4 | 10.973 | 0.031 |
|  |  |  | weighted betweenness centrality of L_pOFC | -11.222 | 0.021 |
|  |  |  | weighted betweenness centrality of L_LBelt | -13.828 | 0.004 |
| **SSRS** | 0.438 | 1.002 | group | -7.184 | <0.001 |
|  |  |  | gender | 2.698 | 0.019 |
|  |  |  | weighted betweenness centrality of R_PIT | 1.250 | <0.001 |

Abbreviations: CCSQ, Childhood Chronic Stress Questionnaire；SSRS, Social Support Rating Scale; R, right；L, left; VIP, Ventral IntraParietal Complex; OFC, Orbital Frontal Complex; 1, Area 1; 6mp, Area 6mp; FOP4, Frontal Opercular Area 4; pOFC, posterior OFC Complex; Lbelt, Lateral Belt Complex; PIT, Posterior InferoTemporal Complex.

### Table S7. Group × sex interaction effects on brain network metrics

| **Metric** | **β** | **p Value（FDR corrected）** |
| --- | --- | --- |
| Small-Worldness | 0.022 | 0.496 |
| Global efficiency | 0.016 | 0.700 |
| Network diameter | -0.418 | 0.965 |
| Network radius | -0.527 | 0.523 |
| Degree of R_A5 | 3.426 | 0.904 |
| Degree of R_MST | 2.255 | 0.965 |
| Weighted local efficiency of L_LBelt | 0.011 | 0.965 |
| Weighted local efficiency of R_OFC | -0.085 | 0.941 |
| Weighted local efficiency of R_VIP | 0.017 | 0.965 |
| Weighted betweenness centrality of L_1 | 155.616 | 0.965 |
| Weighted betweenness centrality of L_13l | 77.95 | 0.863 |
| Weighted betweenness centrality of L_2 | 463.698 | 0.965 |
| Weighted betweenness centrality of L_23c | -672.632 | 0.867 |
| Weighted betweenness centrality of L_3b | -485.578 | 0.965 |
| Weighted betweenness centrality of L_47m | 12.835 | 0.979 |
| Weighted betweenness centrality of L_6a | 29.085 | 0.979 |
| Weighted betweenness centrality of L_6ma | 34.339 | 0.979 |
| Weighted betweenness centrality of L_6mp | -844.957 | 0.751 |
| Weighted betweenness centrality of L_6r | -1548.347 | 0.385 |
| Weighted betweenness centrality of L_8BL | -124.336 | 0.979 |
| Weighted betweenness centrality of L_9p | -315.211 | 0.875 |
| Weighted betweenness centrality of L_FOP4 | 78.646 | 0.979 |
| Weighted betweenness centrality of L_LBelt | 64.978 | 0.965 |
| Weighted betweenness centrality of L_LO2 | 36.305 | 0.965 |
| Weighted betweenness centrality of L_PGi | 364.999 | 0.800 |
| Weighted betweenness centrality of L_PHA2 | -5.238 | 0.965 |
| Weighted betweenness centrality of L_RI | 377.174 | 0.816 |
| Weighted betweenness centrality of L_TPOJ2 | -26.722 | 0.965 |
| Weighted betweenness centrality of L_V3B | 107.471 | 0.965 |
| Weighted betweenness centrality of L_V8 | -106.743 | 0.898 |
| Weighted betweenness centrality of L_pOFC | -286.447 | 0.904 |
| Weighted betweenness centrality of R_10r | 243.943 | 0.800 |
| Weighted betweenness centrality of R_24dv | 198.624 | 0.850 |
| Weighted betweenness centrality of R_44 | 257.176 | 0.766 |
| Weighted betweenness centrality of R_5m | 7.255 | 0.965 |
| Weighted betweenness centrality of R_6ma | -327.567 | 0.949 |
| Weighted betweenness centrality of R_7PC | 12.109 | 0.965 |
| Weighted betweenness centrality of R_8Ad | -50.428 | 0.965 |
| Weighted betweenness centrality of R_8Av | 62.16 | 0.904 |
| Weighted betweenness centrality of R_PIT | 34.34 | 0.965 |
| Weighted betweenness centrality of R_V4 | -122.9 | 0.875 |
| Weighted eigenvector centrality of R_pOFC | 0.001 | 0.965 |
| Weighted pagerank centrality of L_FST | ＜0.001 | 0.774 |
| Weighted pagerank centrality of R_MST | ＜0.001 | 0.979 |
| Weighted pagerank centrality of R_V2 | ＜0.001 | 0.979 |

Abbreviations: R, right；L, left；A5, Auditory 5 Complex; MST, Medial Superior Temporal Area; Lbelt, Lateral Belt Complex; OFC, Orbital Frontal Complex; VIP, Ventral IntraParietal Complex; 1, Area 1; 13l, Area 13l; 2, Area 2; 23c, Area 23c; 3b, Primary Sensory Cortex; 47m, Area 47m; 6a, Area 6 anterior; 6ma, Area 6m anterior;6mp, Area 6mp; 6r, Rostral Area 6; 8BL, Area 8B Lateral; 9p, Area 9 Posterior; FOP4, Frontal Opercular Area 4; LO2, Area Lateral Occipital 2; PGi, Area PGi; PHA2, ParaHippocampal Area 2; RI, RetroInsular Cortex; TPOJ2, Area TemporoParietoOccipital Junction 2; V3B, Area V3B; V8, Eighth Visual Area; pOFC, posterior OFC Complex; 10r, Area 10r; 24dv, Ventral Area 24d; 44, Area 44; 5m, Area 5m; 7PC, Area 7PC; 8Ad, Area 8Ad; 8Av, Area 8Av; PIT, Posterior InferoTemporal Complex; V4, Fourth Visual Area; FST, Area FST; V2, Second Visual Area.

### Figure S1. The brain region showed significantly higher weighted eigenvector centrality in adolescents with MDD compared with HCs.


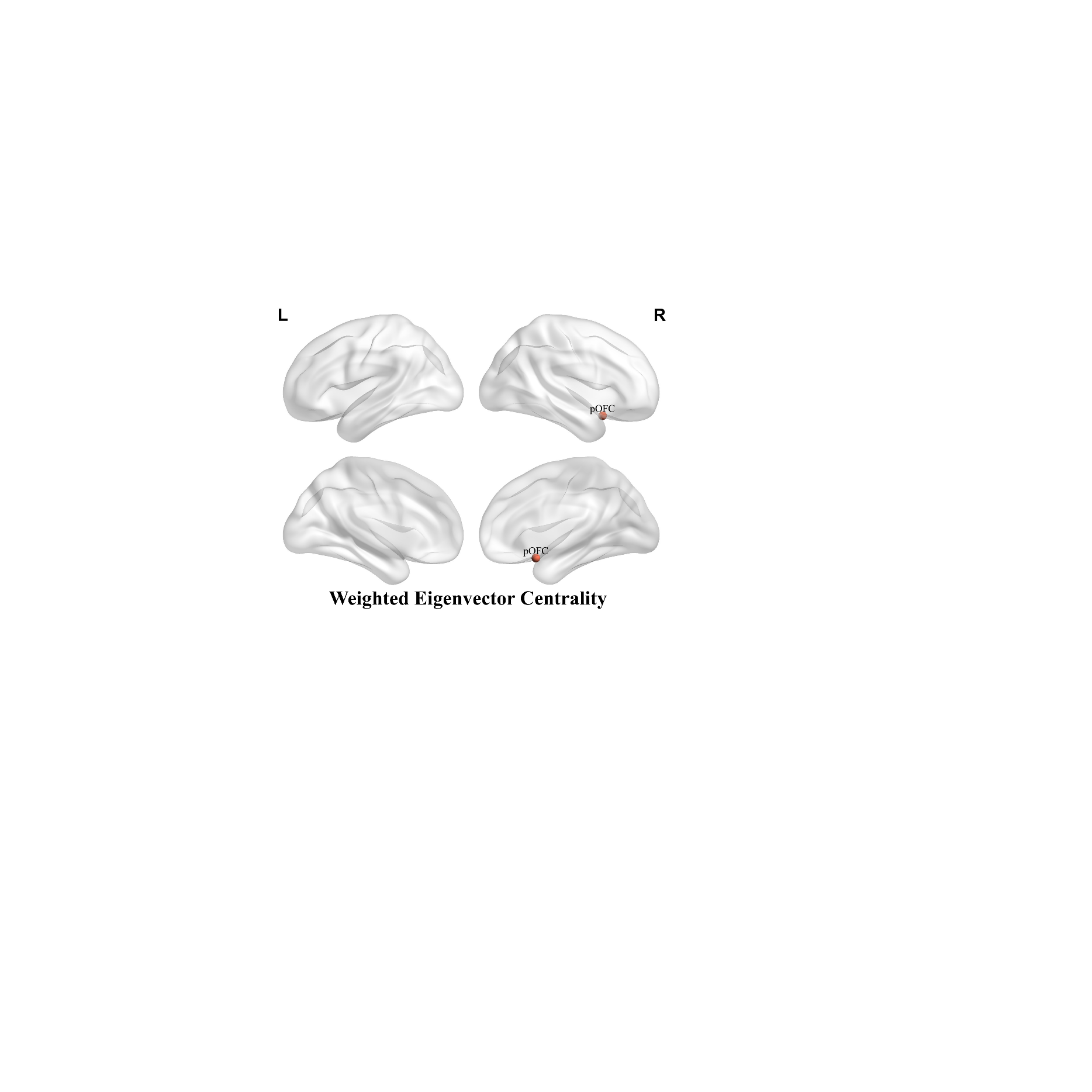


Abbreviations: R, right；L, left ; pOFC, posterior OFC Complex.
